# LncRNApred: Classification of Long Non-Coding RNAs and Protein-Coding Transcripts by the Ensemble Algorithm with a New Hybrid Feature

**DOI:** 10.1371/journal.pone.0154567

**Published:** 2016-05-26

**Authors:** Cong Pian, Guangle Zhang, Zhi Chen, Yuanyuan Chen, Jin Zhang, Tao Yang, Liangyun Zhang

**Affiliations:** Department of Mathematics, College of Science, Nanjing Agricultural University, Nanjing, Jiangsu, People’s Republic of China; CSIR Institute of Genomics and Integrative Biology, INDIA

## Abstract

As a novel class of noncoding RNAs, long noncoding RNAs (lncRNAs) have been verified to be associated with various diseases. As large scale transcripts are generated every year, it is significant to accurately and quickly identify lncRNAs from thousands of assembled transcripts. To accurately discover new lncRNAs, we develop a classification tool of random forest (RF) named LncRNApred based on a new hybrid feature. This hybrid feature set includes three new proposed features, which are MaxORF, RMaxORF and SNR. LncRNApred is effective for classifying lncRNAs and protein coding transcripts accurately and quickly. Moreover,our RF model only requests the training using data on human coding and non-coding transcripts. Other species can also be predicted by using LncRNApred. The result shows that our method is more effective compared with the Coding Potential Calculate (CPC). The web server of LncRNApred is available for free at http://mm20132014.wicp.net:57203/LncRNApred/home.jsp.

## Introduction

More and more studies have indicated that protein coding genes account for less than 2% of the mammalian genome over the past decades[1–11]. A huge mass of genome that was previously regarded as “dark matter” is transcribed to non-coding RNAs (ncRNAs) [12–16]. Moreover, an increasing number of studies shows that ncRNAs have crucial and essential regulatory functions, even if it doesn’t encode proteins [17]. According to the size of transcripts, ncRNAs fall into two categories, short and long ncRNAs (lncRNAs). Short ncRNAs roughly consist of small nucleolar RNAs (snoRNAs), microRNAs (miRNAs), piwi-interacting RNAs (piRNAs), short-interfering RNAs (siRNAs) and short hairpin RNAs (shRNAs) [18–21]. In general, the length of short ncRNAs is shorter than 200 nt. In contrast the length of lncRNAs is longer than 200 nt [22]. As the major part of eukaryotic transcriptomes, lncRNAs have been verified to be associated with various diseases like cancers[23–30], heart failure [31–34], AIDS [35–41]. LncRNADisease database was constructed by Chen et al. [42], and contains more than 1000 lncRNA-disease entries, including 321 lncRNAs and 221 diseases from nearly 500 publications. Therefore, the identification and annotation of lncRNAs are crucial steps for understanding various regulatory mechanisms.

With the development of current experimental technology, a large number of lncNRAs have been annotated in the transcriptome. However, experimental methods have certain limits, such as the poor expression of most lncRNAs and the difficulty of enormous experimental data analysis [15,43]. Thus, it is essential to develop computational methods to identify lncRNAs from the transcriptome accurately and quickly.

There are many methods to identify ncRNAs [44–55]. For instance, Liu et al. introduced a tool called CONC (coding or non-coding) based on support vector machines (SVM) to classify transcripts according to a hybrid feature set [56]. This feature set consists of alignment entropy, amino acid composition, predicted percentage of exposed residues, predicted secondary structure content, number of homologs from database searches, compositional entropy and peptide length. However, CONC is slow for abundant datasets, and its web server is not available. Moreover, the outputs of CONC does not provide related information. Thus, Lei et al. developed a online software called Coding Potential Calculator (CPC) to identify the protein-coding potential of transcripts based on six biologically meaningful sequence features [57]. Compared with CONC, CPC is more accurate and run faster. It also has a more friendly web interface. Lin et al. present a software named PhyloCSF to distinguish protein coding by analyzing a multispecies nucleotide sequence alignment. It is a method of comparative genomics [58]. Their results indicate PhyloCSF is applicable for evaluating the protein-coding potential of transcript models or individual exons. Lei Sun et al. [59] develop a tool named LncRScan-SVM by integrating features derived from gene structure, transcript sequence, potential codon sequence and conservation. Kun Sun et al. [60] use one conservation, two Open Reading Frame (ORF) and seven nucleotide sequence features to construct a support vector machine classifier (iSeeRNA) for the identification of long intergenic non-coding RNAs (lincRNAs). Liguo Wang et al. [61] build a tool named Coding Potential Assessment Tool (CPAT), which can rapidly identify coding and non-coding transcripts. CPAT uses a logistic regression model built with four sequence features: open reading frame coverage hexamer usage bias, Fickett TESTCODE statistic and open reading frame size. However, the above tools are not suitable for classifying lncRNAs, which contain long putative Open Reading Frame (ORF) or short protein-like sub-sequences [62,63]. To overcome the challenge, Liang Sun et al. [64] develop the Coding-Non-Coding Index (CNCI) software, a powerful tool, by profiling adjoining nucleotide triplets (ANT), to effectively recognize protein-coding and non-coding sequences.

In this paper, we introduce a generalized classifier based on an integrated algorithm called random forest (RF) to distinguish lncRNAs from protein-coding transcripts. Besides, we propose three new features, which are MaxORF, RMaxORF and SNR. A new hybrid feature set with 89 dimension can be formed by combining 86 sequence features and the three new features just mentioned together. The results show that the first three important features are MaxORF, SNR and RMaxORF. At the same time, we develop a user-friendly web server named LncRNApred and compare the LncRNApred with Coding Potential Calculator(CPC). LncRNApred demonstrates better performance compared with CPC.

## Materials and Methods

### Datasets

The NONCODE version 3.0 [65] (http://www.noncode.org/NONCODERv3/) currently contains 33665 non-redundant lncRNA sequences of human. In this paper, 33665 lncRNAs are selected as positive samples. For the negative samples, protein-coding transcripts are extracted from UCSC database [66] (http://hgdownload.soe.ucsc.edu/downloads.html), from which 38268 mRNAs can be obtained. After removing the mRNAs with length of <20000 and >200, 38229 mRNA sequences are retained.

In order to avoid over-fitting, some redundant samples should be removed. Therefore, we select 2033 lncRNAs and 2031 mRNAs from 33665 lncRNAs and 38229mRNAs respectively as the training dataset by Self Organizing Feature Map (SOM) [67]. These training samples can effectively describe the whole data. The remaining samples are used to assess our model.

In order to test the generalization of our RF classifier, 35851 lncRNAs and 27728 mRNAs of mouse are obtained from the database of NONCODE version 3.0 and UCSC database respectively [65,66]. In addition, 2551 lncRNAs of other species are downloaded from NONCODE version 3.0. Repetitive sequences and those with other letters except for 'A', 'a', 'C', 'c', 'G', 'g', 'T', 't', 'U', 'u' are removed. The remaining 2113 lncRNAs of other species and above samples of mouse are also used to evaluate our classifier.

### The selection of training samples

The accuracy of a RF classifier depends highly on the selection of training samples. So we should select representative samples to construct training dataset. In this paper, we use a clustering method to obtain representative samples. In order to find an appropriate clustering method, we analysis four different cases: (1) k-means clustering (2) hierarchical clustering (3) SOM (Self Organizing Feature Map) clustering (4) non-clustering. In the first three cases, we use three different clustering methods to select 2000 lncRNAs from 33665 lncRNAs and 2000 mRNAs from 38229 mRNAs as the training dataset. In the fourth case, we randomly select 2000 lncRNAs from 33665 lncRNAs and 2000 mRNAs from 38229 mRNAs as the training dataset of RF. Therefore, four RF models can be constructed respectively. As shown in Table 1, the classification performance after the pretreatment of clustering is better than that without the pretreatment of clustering. Besides, the results also show that SOM clustering algorithm outperforms the other three cases. According to the above discussion, Self Organizing Feature Map (SOM) is used to select representative samples in our paper.

**Table 1:** The classification performance after the pretreatment of clustering.

Method	S_p _(%)	S_n _(%)	ACC (%)
RF	91.2	90.4	90.8
K-means+RF	92.4	91.2	91.8
Hierarchical+RF	92.6	91.4	92.0
**SOM+RF**	**93.4**	**92.5**	**92.9**

SOM is a type of Artificial Neural Network (ANN). In 1990, Teuvo Kohonen proposed SOM [67] and effectively used it to classify input vectors according to the way they are grouped in the input space. SOM is different from other artificial neural networks as they apply competitive learning as opposed to error-correction learning (such as Back Propagation Artificial Neural Network), and in the sense that they use a neighborhood function to preserve the topological properties of the input space.

Like most artificial neural networks, SOMs operate in two modes: training and mapping. "Training" builds the map using input examples (a competitive process), while "mapping" automatically classifies a new input vector.

**Fig 1:**
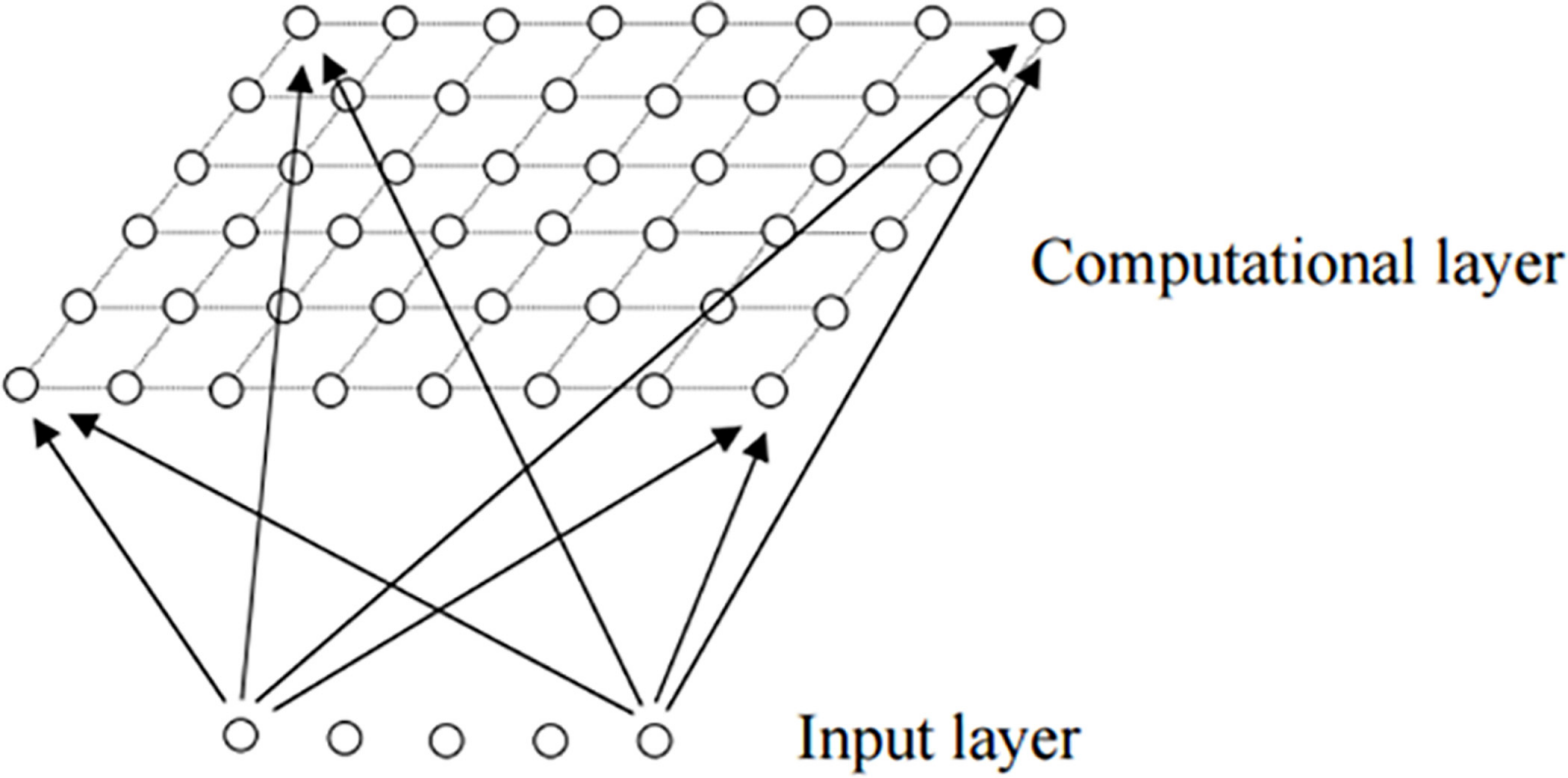
Two dimensional SOM neural network model.

A SOM consists of components called neurons. Associated with each node is a weight vector of the same dimension as the input data vector. The self-organizing map describes a mapping from a higher-dimensional input space to a lower-dimensional map space. The procedure for placing a vector from data space onto the map is to find the node with the closest (smallest distance metric) weight vector to the data space vector. Fig 1 describes two dimensional SOM neural network model. All neurons in the competition layer are fully connected.

The main SOM learning algorithm can be described as follows:

Let *X* = [x_1_,x_2_,⋯,x_*m*_], be the input vector. We construct two-dimensional network with n output node. Set *w*_*ij*_ be the weight vector connecting the *i*th input node and the *j*th output nodes.

(1) Initialization of weights.

The weights (*w*_*ij*_) should be initialized randomly. The value of every weight must be different.

(2) Calculate the distance between the input vector and weight vector.

$$d_{j}=\sum_{i=1}^{m}(x_{i}(t)-w_{ij}(t){)}^{2}.$$ (1)

*x*_*i*_(*t*) represents the value of input vector *x* at time t.

(3) Select the winning neuron *i*(*x*).

Select the nearest unit as winner. The neuron *i* is the winning neuron.

$$i\left(x\right)=\underset{j}{\min}(d_{j}).$$ (2)

(4) Adjust the connection weight vector of the output node.

Update weight vector of the SOM according to the update function:

$$w_{ij}(t+1)=w_{ij}(t)+\eta (t)h_{j,i(x)}(t)(x(t)-w_{ij}(t)).$$ (3)

where *η*(*t*) is a learning efficiency function. To ensure the convergence of the learning process, *η*(*t*) is monotonically decreasing. *h*_*j*,*i*(*x*)_ is a neighborhood function of the winning neuron.

(5) Repeat the step (2) to (4), and update the learning parameters, until a certain stopping criterion is met.

We use the following steps to select the training dataset.

Given a dataset *Q* = {*x*_*i*_ | *x*_*i*_ ∈ *R*^*n*^, *i* = 1,…, *N*}, *K* is the number of neurons in the competitive layers.

Step 1: The *N* samples are imported to the input layer of SOM.

Step 2: Calculate the number of training samples for every neurons in the competitive layers and record them as *w* = [*w*_1_,*w*_2_,⋯,*w*_*K*_],

Step 3: Let *L* be the number of training dataset. Randomly select *O*_*i*_ samples from the *i*th neuron as the training samples. *O*_*i*_ can be calculated by the following formula

$$O_{i}=\left\lceil \frac{w_{i}}{N}\times L\right\rceil ,$$ (4)

where ⌈A⌉ rounds the element of A to the nearest integers greater than or equal to A.

Step4: The (*O*_1_+*O*_2_+⋯+*O*_*K*_) samples of training dataset can be obtained.

**Fig 2:**
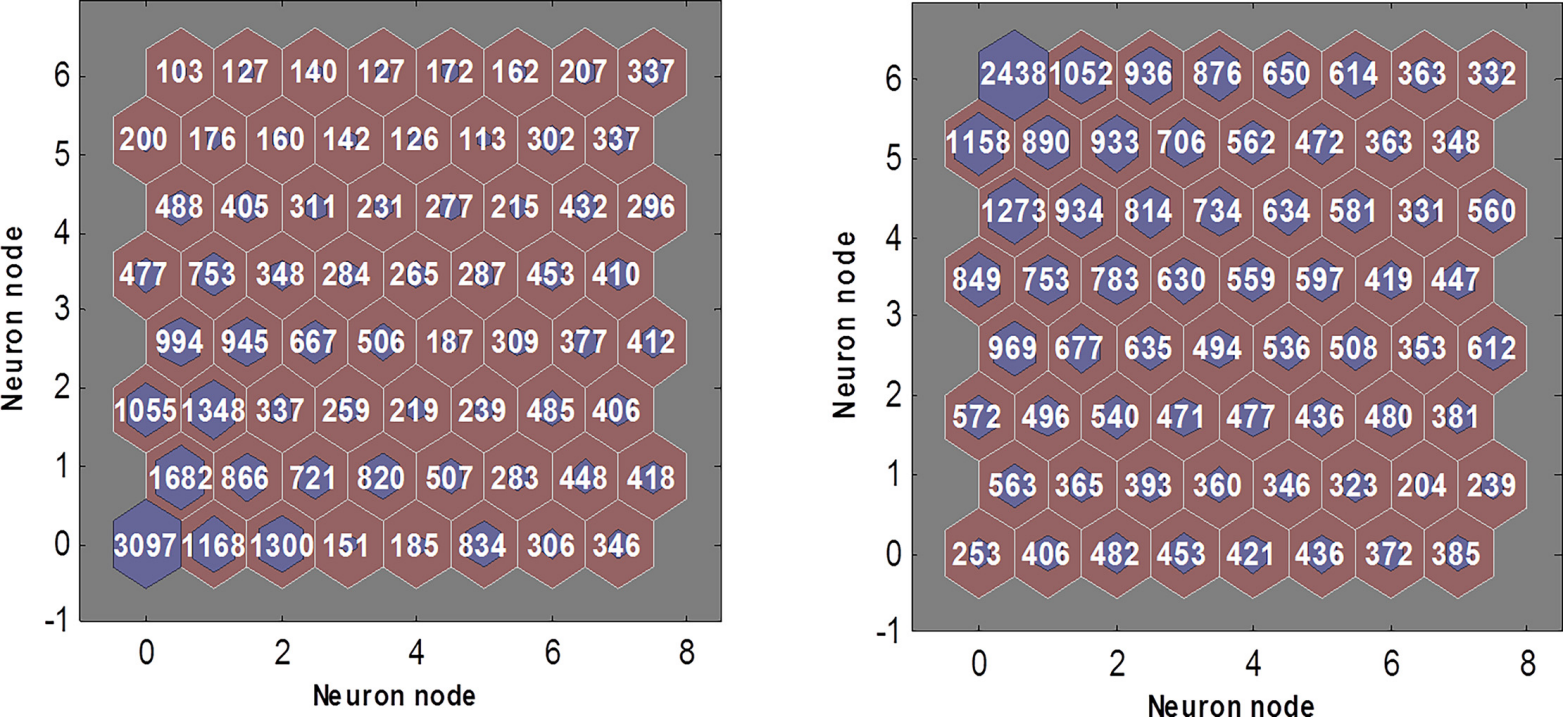
The result of SOM clustering. The left side represents the distribution in the 64 neurons of lncRNAs. Every digit of the hexagon is the number of lncRNAs which belong to one class. The right side represents the distribution in the 64 neurons of mRNAs, and every digit of hexagon is the number of mRNAs which belong to one class.

In this study, we choose 8 × 8 neurons in the competitive layers and 2000 training samples. Fig 2 shows the distribution in the 64 neurons of lncRNAs or mRNAs. Each hexagon represents one neuron and there are 64 neurons in total. Every digit inside the hexagon is the number of lncRNAs (or mRNAs) which belong to the corresponding neuron. All neurons in the competition layer are fully connected. We use above steps to choose training samples. For example, neuron node in the lower right corner of Fig 2 is 385 and the total number of mRNAs is 38229. Thus, we should randomly select 2000×⌈385/38229⌉ samples from that neuron node. The final number of mRNA training samples *N*_*mRNA*_ and LncRNA training samples *N*_ln *cRNA*_ are as follows:

$$N_{mRNA}=2000\times (\left\lceil \frac{2438}{38229}\right\rceil +\left\lceil \frac{1052}{38229}\right\rceil +\cdots +\left\lceil \frac{372}{38229}\right\rceil +\left\lceil \frac{385}{38229}\right\rceil )=2031$$ (5)

$$N_{\ln cRNA}=2000\times (\left\lceil \frac{103}{30740}\right\rceil +\left\lceil \frac{127}{30740}\right\rceil +\cdots +\left\lceil \frac{306}{30740}\right\rceil +\left\lceil \frac{346}{30740}\right\rceil )=2033$$ (6)

### Feature

#### Signal to noise ratio (SNR)

Let *s*[*n*] be a sequence of length *N*. Let *I* = {*A*,*G*,*C*,*T*}, for any *b* ∈ *I*.

$$u_{b}[n]=\{\begin{array}{c} 1\;,\;S[n]=b\\ 0,\;S[n]\neq b \end{array}\;n=0,1,2,\cdots ,N-1$$ (7)

There are four binary indicator sequence {u_*b*_[*k*]}, *b* ∈ *I*, which is called Voss mapping [68]. For instance, given a DNA sequence as follows:

$$5^{'}\ldots ATCTCACTGGT\ldots 3^{'}$$

the Voss mapping of this DNA sequence can be represented as

$$\begin{array}{l} u_{T}=\{\ldots 0,1,0,1,0,0,0,1,0,0,1\ldots \},\;u_{A}=\{\ldots 1,0,0,0,0,1,0,0,0,0,0\ldots \},\\ u_{C}=\{\ldots 0,0,1,0,1,0,1,0,0,0,0\ldots \},\;u_{G}=\{\ldots 0,0,0,0,0,0,0,0,1,1,0\ldots \}. \end{array}$$

Using Discrete Fourier Transform (DFT) on the indicator sequences respectively, we get for *b* ∈ *I*,

$$U_{b}[k]=\sum_{n=0}^{N-1}u_{b}[n]e^{-i\frac{2\pi nk}{N}},\;k=0,1,\cdots ,N-1.$$ (8)

There are four complex sequences ({*U*_*b*_[*k*]}, *b* ∈ *I*) in total. The power spectrum of the whole sequence is defined as {*P*[*k*]}:

$$P[k]={\left|U_{A}[k]\right|}^{2}+{\left|U_{T}[k]\right|}^{2}+{\left|U_{G}[k]\right|}^{2}+{\left|U_{C}[k]\right|}^{2},\;k=0,1,\cdots ,N-1$$ (9)

**Fig 3:**
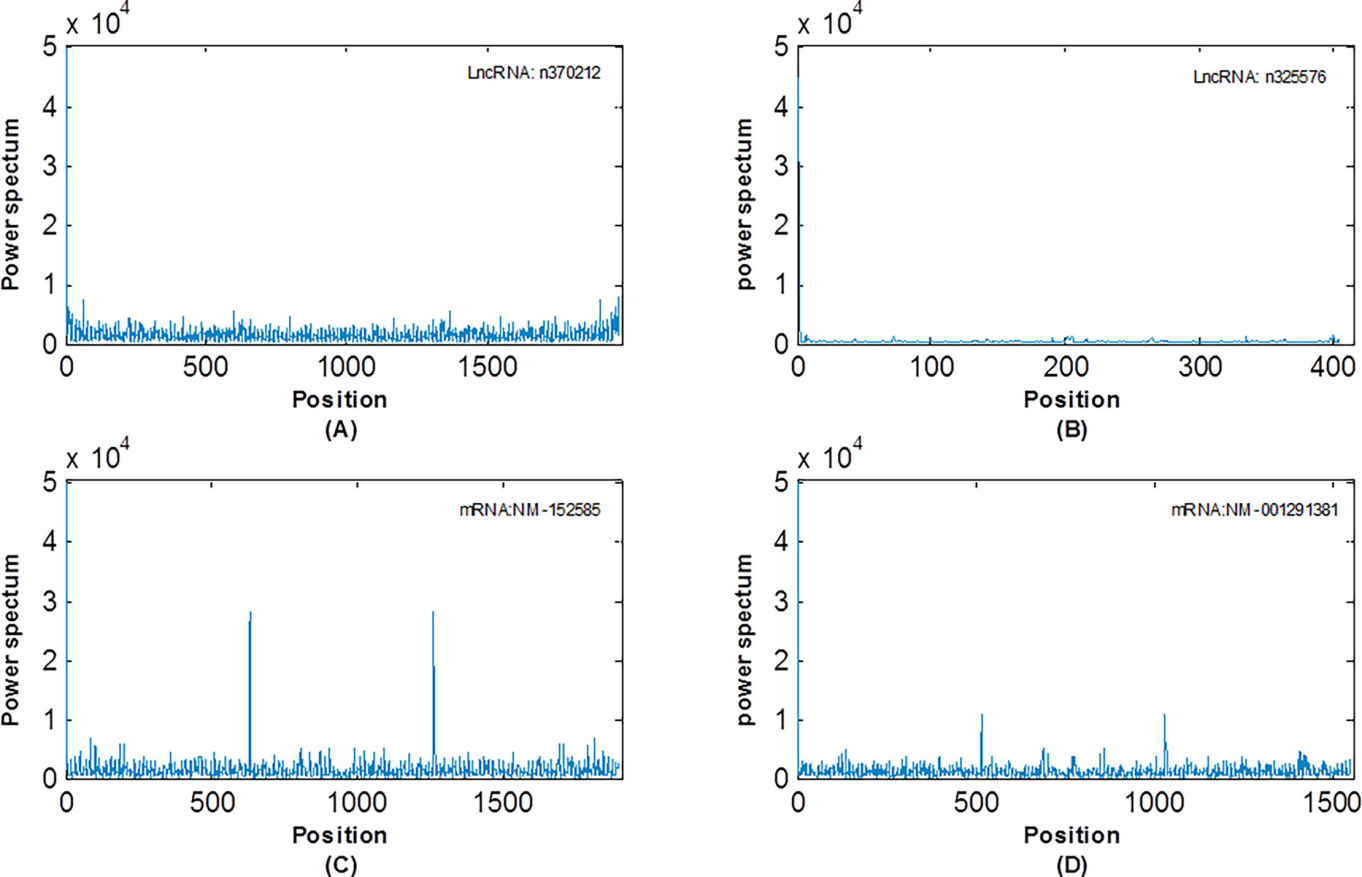
Power spectrum of mRNAs and lncRNAs. (A) and (B) represent the power spectrum of two different lncRNAs, and (C) and (D) represent power spectrum of two different mRNAs.

Given a sequence, the power spectrum curve can be obtained by (9). In Fig 3, an obvious peak appeared at *N*/3 in the power spectrum curve of the mRNA sequence, while there is no peak in the lncRNA sequence. This statistical phenomenon is known as the period-3 behavior [69]. It was proved that the 3-base periodicity is mainly caused by the unbalanced nucleotide distributions in a DNA sequence [70,71,72,73]. The nucleotide distribution in the three codon positions is unbalanced in a coding sequence, while in a non-coding sequence, the nucleotides distribute uniformly in the three codon positions. The main reason of this phenomenon is that proteins prefer special amino acid and thus nucleotide usage in a coding region is highly biased.

Signal to noise ratio (SNR) is defined as following:

$$SNR=\frac{P[\frac{N}{3}]}{\bar{E}}$$ (10)

$$\bar{E}=\frac{\sum_{k=0}^{N-1}P[k]}{N},$$ (11)

where $\bar{E}$is the mean of the total power spectrum of the whole sequence [69].

**Fig 4:**
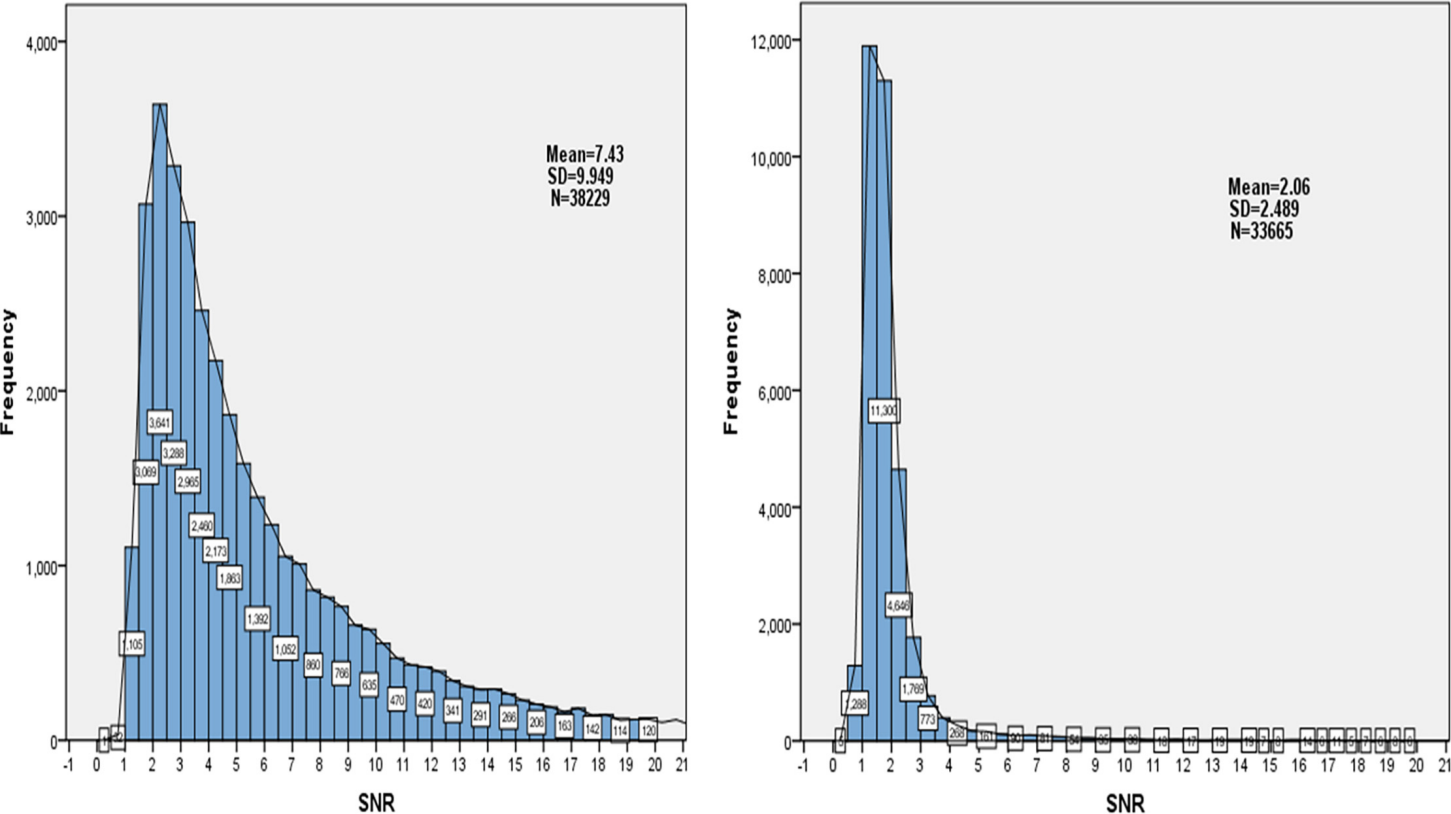
The distribution of SNR. The left side represents the SNR distribution of 38229 mRNAs, and the right side represents SNR distribution of 33665 lncRNAs.

SNR not only shows the relative height of the spectrum peak, but also reflects the 3-periodic property. As shown in Fig 4, the white boxes on the bar graph represent the number of mRNA (or lncRNA) in each bar. The mean of SNR of mRNAs and lncRNAs are 7.43 and 2.06 respectively. Besides, we calculate that 72.7% (24488/33665) SNR of lncRNAs are less than 2. On the contrary, 89% (34020/38229) SNR of mRNAs are greater than 2. The P-value is 7.3123e-115 by Student’s t-test. The result shows that there are obvious differences in the SNR between the positive samples and negative samples. Therefore, SNR can be used to distinguish lncRNA and mRNA as an important feature.

#### Open reading frame (ORF)

Compared with long non-coding transcripts, protein coding transcripts are more likely to have a long ORF. Therefore, we select two ORF features to distinguish lncRNAs and protein coding transcripts. One is the length of the longest ORF (MaxORF) in the three forward frames, and the other is the normalized MaxORF (RMaxORF).

$$RMaxORF=\frac{MaxORF}{L},$$ (12)

where *L* is the length of sequence.

#### Sequence features

In this work, 4 1-mer strings, 16 *2-*mer strings and 64 *3-*mer strings are used to identify lncRNA and mRNA. Besides, the length of sequence (Length) and (G+C)% are selected as two sequence features.

### Feature selection

For a lncRNA sequence or mRNA sequence, we combine the 1 dimensional SNR feature, 2 dimensional ORF features and 86 dimensional sequence features to get a hybrid feature vector with 89 dimension. However, not every feature contributes to the classification accuracy. Golub et al. [74] use the feature score criterion (FSC) to calculate the score of each feature, and rank them in descending order. The first p features are selected as the information features. Setting p<n (n is the dimension of features), we need to determine the optimal p value by the experimental results. As shown in Table 2, the second line represents the performance of RF model with the top 5 features. The Sensitivity (Sn) and Specificity (Sp) are 91.2% and 90.2% respectively. The experimental results show that the performance of RF model is relatively stable while p>30. At the same time, the accuracy of RF classifier reaches maximum when p = 30, and the Sensitivity (Sn) and Specificity (Sp) are 93.4% and 92.5% respectively. Therefore, we choose p = 30 as the information feature set of RF classifier.

**Table 2:** Effect of the number of features on the classification accuracy rate of V-ELM.

Number of features (p)	Sn (%)	Sp (%)
4	91.0	89.1
5	91.2	90.2
10	91.5	90.7
15	92.4	90.9.
20	92.6	91.0
25	93.1	91.6
**30**	**93.4**	**92.5**
35	93.2	92.1
40	93.1	92.0
45	93.4	92.2
50	93.3	92.3
55	93.4	92.1
60	93.2	92.4
86	92.9	92.3

On the premise of the optimal classification accuracy, the minimum value of p is selected. The score of each feature can be obtained by the following formula.

$$FSC(g_{i})=|\frac{\mu_{i}^{+}-\mu_{i}^{-}}{\sigma_{i}^{+}+\sigma_{i}^{-}}|,$$ (13)

where $\mu_{i}^{+}$($\mu_{i}^{-}$) and $\sigma_{i}^{+}$($\sigma_{i}^{-}$) are the mean and standard deviation respectively of the feature of *g*_*i*_ in the positive (negative) class samples. The higher the *FSC* score is, the stronger classification ability the feature has.

**Fig 5:**
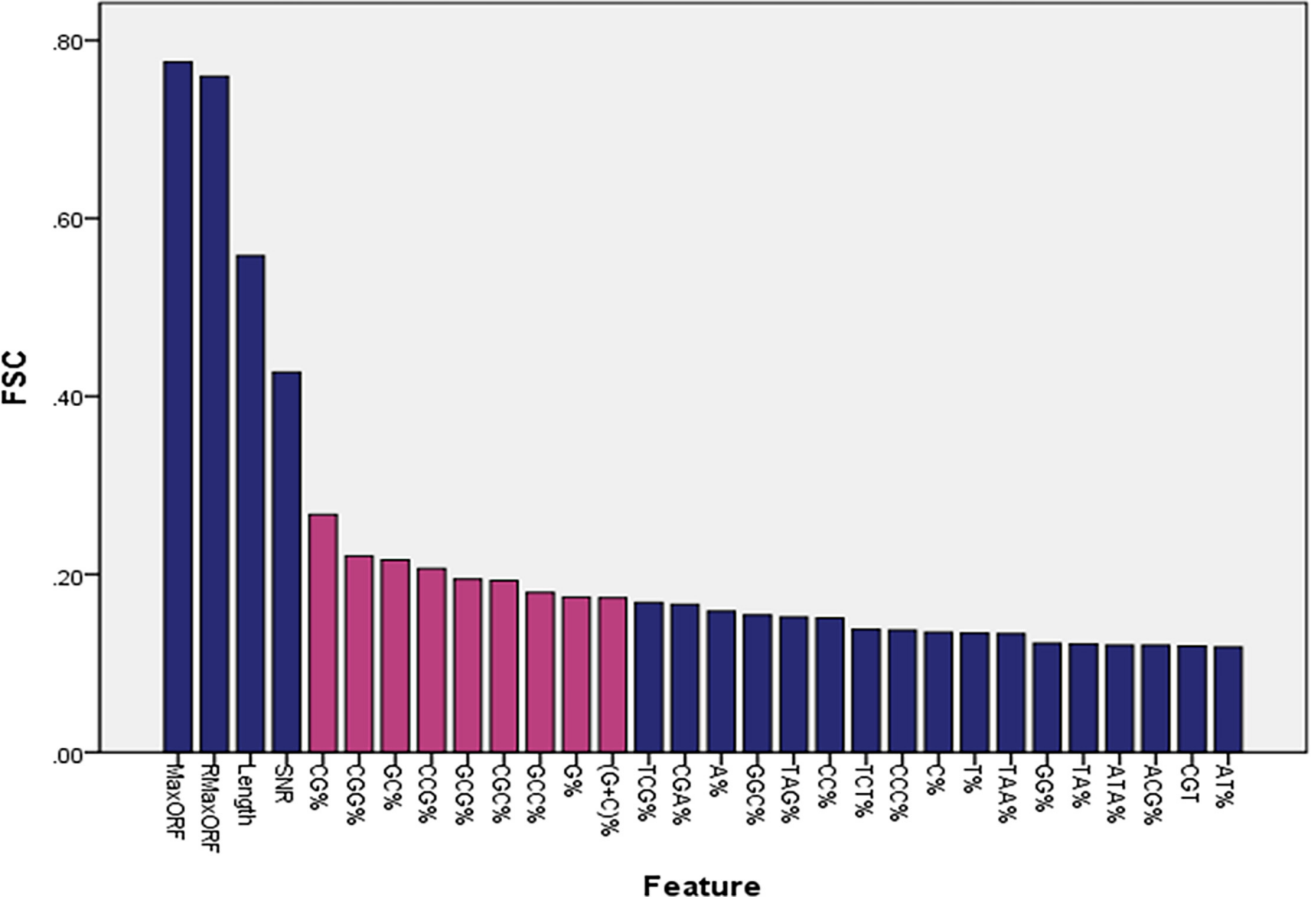
The bar chart shows the top 30 of FSC score.

**Fig 6:**
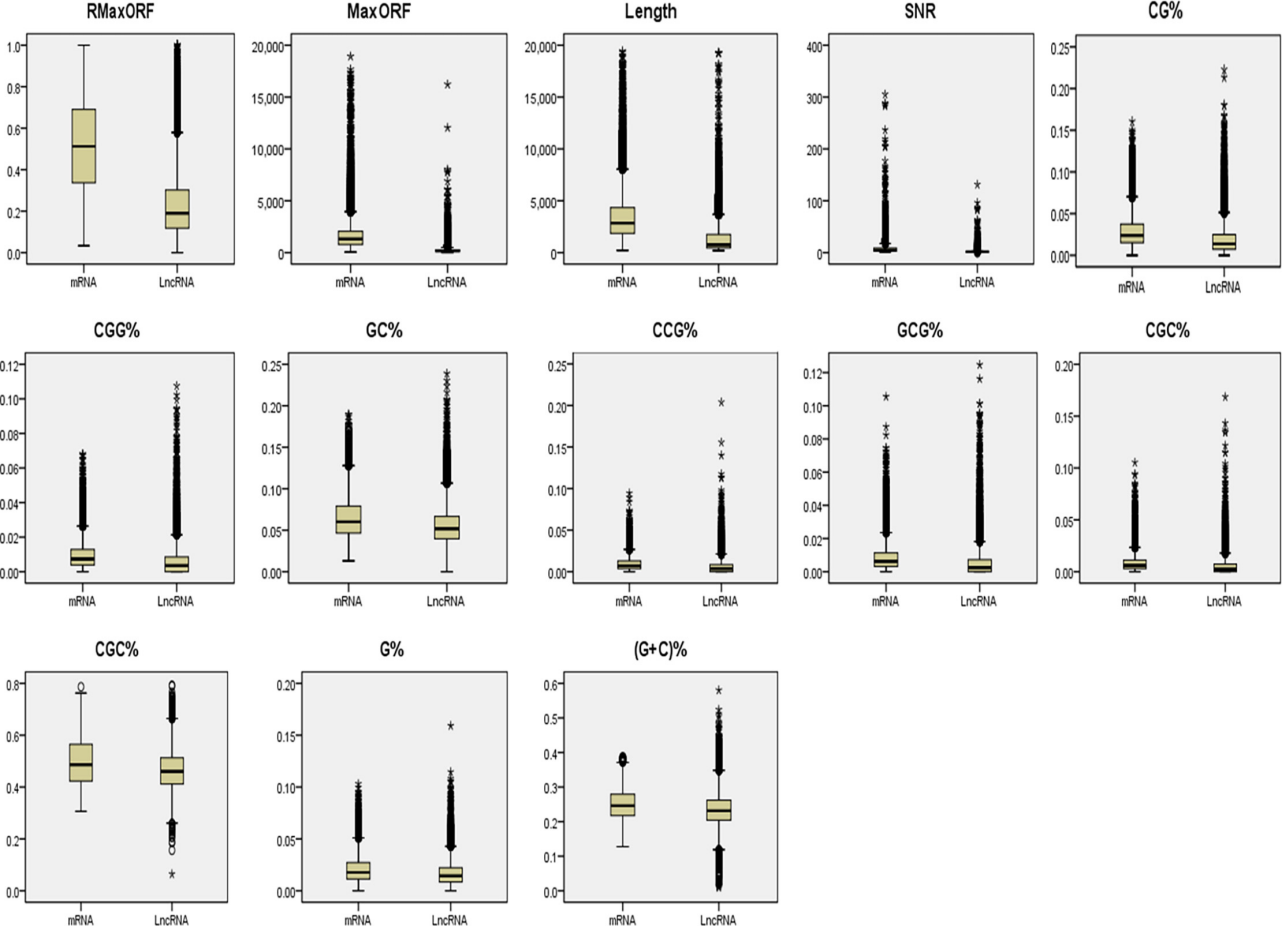
Boxplots of the top 13 features: MaxORF, RMaxORF, SNR, Length, CG%, CGG%, GC%, CCG%, GCG%, CGC%, GCC%, G% and (G+C)%. For each plot, the left side represents the mRNA, and the right side represents lncRNA.

As shown in Fig 5, a set of 30 features from the 89 features was selected by FSC, including MaxORF, RMaxORF, Length, SNR, CG%, CGG%, GC%, CCG%, GCG%, CGC%, GCC%, G%, (G+C)%, TCG%, CGA%, A%, GGC%, TAG%, CC%, TCT%, CCC%, C%, T%, TAA%, GG%, TA%, ATA%, ACG%, CGT%, and AT%. We find that the FSC differences of 30 features between lncRNAs and mRNAs are apparent, especially the features of MaxORF, RMaxORF, SNR and Length. In addition, except for MaxORF, RMaxORF, SNR and Length, the Sn and Sp for top four features are 91% and 89.1% respectively. We mark the following 8 features (CG%, CGG%, GC%, CCG%, GCG%, CGC%, GCC%, G%, (G+C)%) in red. We find that these features only relate to the nucleotide of ‘C’ or ‘G’. In order to visualize the spread of the lncRNAs and mRNAs for the top 13 features, graphical boxplots are shown in Fig 6.

### Prediction System Assessment

For a prediction problem, a classifier can classify an individual instance into the following four categories: false positive (*F*_*P*_), true positive (*T*_*P*_), false negative (*F*_*N*_) and true negative (*T*_*N*_). The total prediction accuracy (*ACC*), Specificity (*S*_*p*_), Sensitivity (*S*_*n*_) and Mathew’s correlation coefficient (*MCC*) [75] for assessment of the prediction system are given by

$$S_{n}=\frac{T_{P}}{T_{P}+F_{N}}$$ (14)

$$S_{p}=\frac{T_{N}}{T_{N}+F_{P}}$$ (15)

$$ACC=\frac{T_{P}+T_{N}}{T_{P}+T_{N}+F_{P}+F_{N}}\times 100\%$$ (16)

$$MCC=\frac{T_{P}\times T_{N}-F_{P}\times F_{N}}{\sqrt{\left(T_{P}+F_{P})\times (T_{N}+F_{N})\times (T_{P}+F_{N})\times (T_{N}+F_{P}\right)}}$$ (17)

where T_P_ is the number of lncRNAs identified correctly, F_N_ the number of lncRNAs identified incorrectly, T_N_ the number of mRNAs identified correctly, and F_P_ the number of mRNAs identified incorrectly.

## Results and Discussion

### Identification framework for lncRNAs

**Fig 7:**
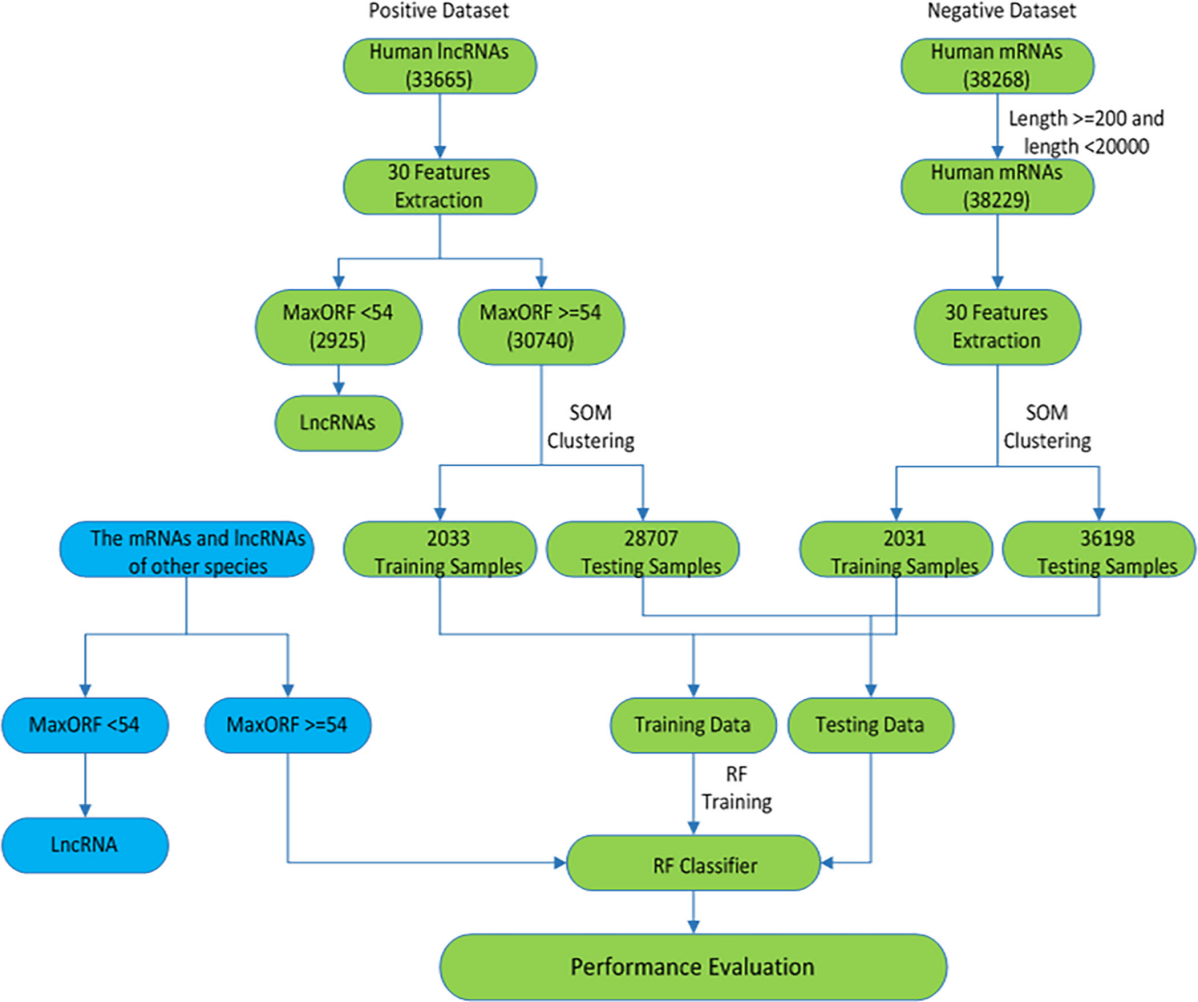
The workflow of lncRNAs identification

The statistical results show that the smallest MaxORF of 38268 mRNAs and 33665 lncRNAs are 54 and 0 respectively. However, the sequences with short ORF usually do not encode proteins. Therefore, we consider that the sequence with MaxORF<54 is regarded as a lncRNA. The workflow of lncRNAs identification model is illustrated in Fig 7. First, 30 dimension features of lncRNAs and mRNAs can be extracted. The lncRNAs with MaxORF>54 are selected as positive dataset. The mRNAs with length ≥200 and length < 20000 are selected as negative dataset. Second, we select representative 2033 lncRNAs and 2031 mRNAs as training samples by the SOM algorithm. The remaining data are used to test the model. Finally, a RF model is constructed based on the training dataset. In addition, we also use other species besides human beings with MaxORF>54 to test our RF classifier except for human. The sequences with MaxORF<54 are directly predicted to be lncRNAs.

### Selection of machine learning algorithms

**Fig 8:**
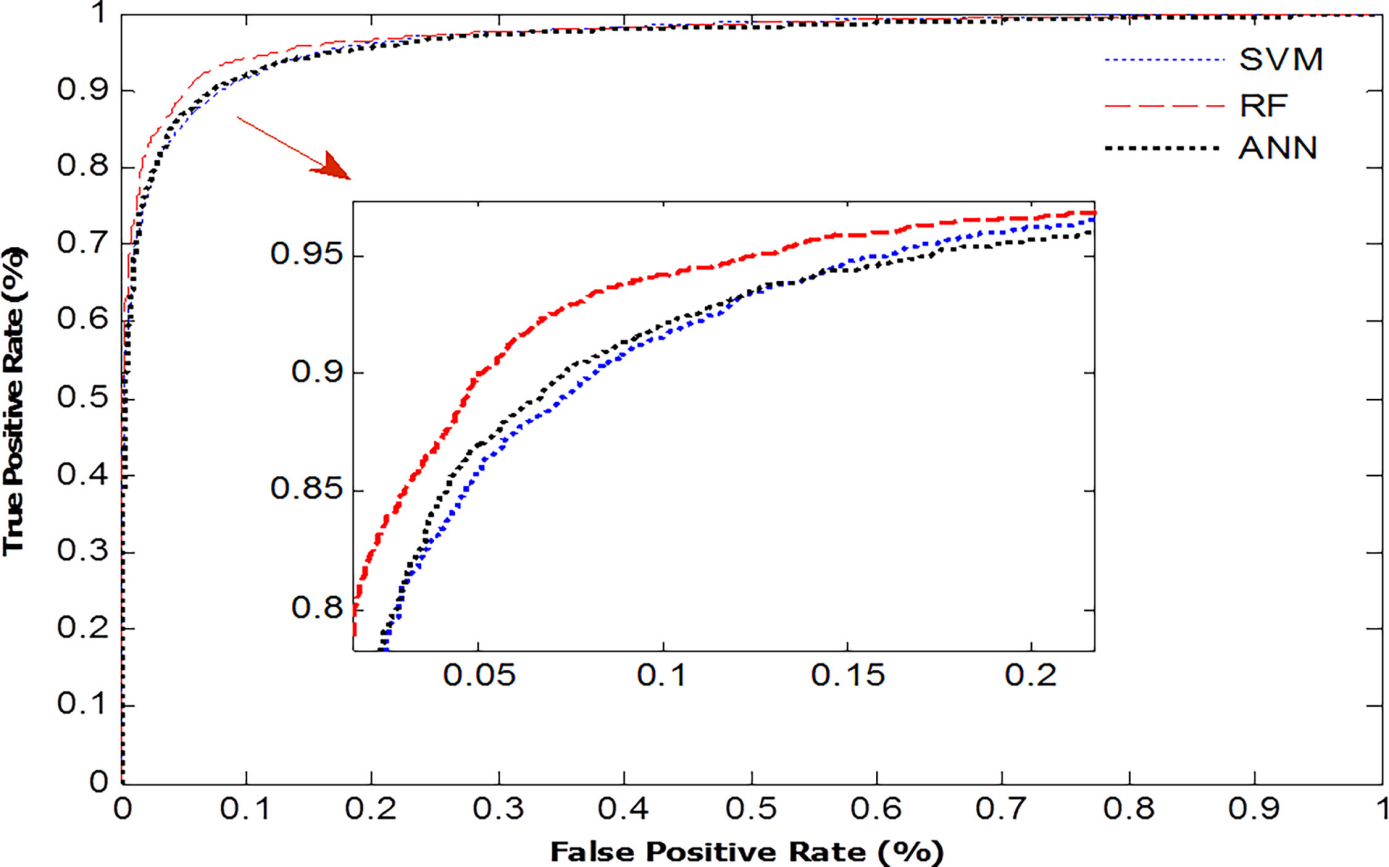
The ROC curves of three different classifiers.

In general, the performance of machine learning algorithms depends on the content of research. Every algorithm has its own advantage. Therefore, we construct three different classifiers by using three algorithms based on the same training dataset to evaluate their performances. The results show that RF algorithm outperforms the two other algorithms for the identification of lncRNAs and mRNAs. To visualize the performance of those three algorithms, we generate ROC curves in Fig 8. The Area Under the Curve (AUC) measures the performance of an algorithm under different thresholds. On average, the AUC of the RF algorithm is about 0.9738. Compared with the AUC of SVM (0.9621) and ANN (0.9649), the robustness of RF model is more obvious. So we use the RF algorithm as the classified model in this work.

The acquiescent parameters C and g of support vector machine (SVM) are 2 and 1 respectively. In order to improve the accuracy of the identification, the optimal parameters of SVM are 1.97062 and 0.061 by the method of the particle swarm optimization (PSO).

In this paper, we use an artificial neural network (ANN) algorithm called voting based extreme learning machine (V-ELM) as the method of comparison. ELM is a kind of quick training algorithms of generalized SLFNs [76,77]. More and more researchers are interested in this method. The hidden layer parameters of SLFNs do not need to be tuned. ELM provides better generalization performance at a much faster learning speed. Because random parameters of the hidden layer nodes are used and remained unchanged during the training process, some samples may be misclassified, especially for those with position close to the classification boundary. In order to avoid this problem and improve the classification performance of ELM, Gao. et al. [78] proposed a new algorithm called voting based extreme learning machine (V-ELM) by incorporating multiple independent ELMs and making decisions with a majority voting method. We select N = 300 as the number of hidden layer nodes in the V-ELM model.

Random forest is an ensemble learning method by constructing multitude of decision trees. This algorithm for inducing a random forest was developed by Leo Breiman and Adele Cutler [79]. Thus "Random Forests" became their trademark. The advantage of a RF algorithm is the robustness provided by random feature selection and the bootstrap aggregating technique [80]. In this paper, we choose N = 300 as the decision trees in our RF model.

### Importance of each feature variable

**Fig 9:**
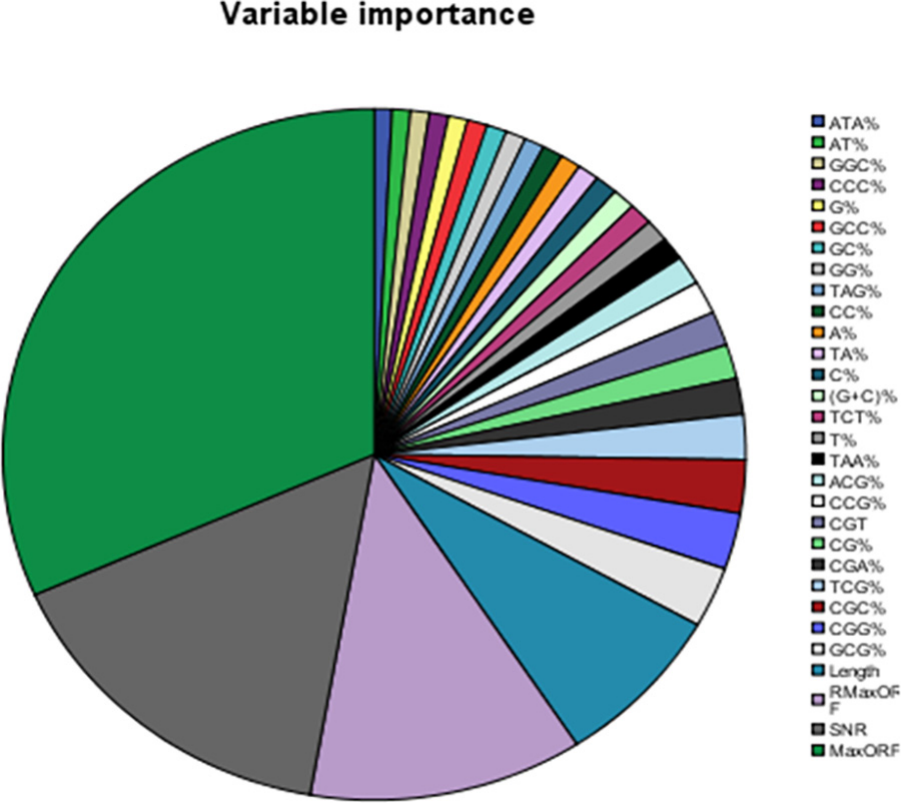
The importance of feature variable.

In order to determine those features which play an important role in the identification of lncRNAs, we use the pie chart based on permutations to show the importance of each feature variable. The RF model can estimate the importance of a feature based on the increases in prediction error when the out-of-bag (OOB) error for that feature is permuted while other features are unchanged. As shown in Fig 9, the size of the area represents the level of the feature importance. We find that the first four important features are MaxORF, SNR, RMaxORF and Length. This chart shows that newly proposed feature can improve the prediction accuracy of lncRNAs.

### Performance evaluation

In this paper, we select 2033 lncRNAs and 2031 mRNAs of human as the training samples by SOM algorithm (S1 and S2 Tables). The remaining 28707 lncRNAs and 36198 mRNAs (S3 and S4 Tables) are used to assess our RF model. As shown in Table 3, the accuracy of lncRNAs and mRNAs are 93.42% (26818/28707) and 92.5% (33483/36198) respectively. Besides, 35851 lncRNAs and 27728 mRNAs (S5 and S6 Tables) of mouse are downloaded from from the database of NONCODE version 3.0.

**Table 3:** The performance of our RF model LncRNApred.

Species	Positive (lncRNAs)	Negative (mRNAs)	Sn	Sp	ACC	MCC
Human	28707	36198	93.42 (26818/28707)	92.5 (33483/36198)	92.96	0.8569
Mouse	35851	27728	95.27 (33699/35851)	93.48 (25921/27728)	94.3	0.8880
Other species	2113	0	97.78 (20668/2113)	0	97.78	0

After removing the lncRNAs of mouse with MaxORF<54, the remaining 35373 lncRNAs and 27728 mRNAs are used to estimate the RF model. Similarly, our RF classifier correctly predicts 95.27% (33699/37373) lncRNAs and 92.7% (25921/27728) mRNAs for the mouse testing dataset.

To further assess the performance of RF model, we download 2113 other species lncRNAs other species from database of NONCODE version 3.0. The last line of Table 3 shows the prediction results of 2113 lncRNAs from other species. The accuracy is 97.78% (2066/2113). These results further indicate the high accuracy of RF classifier for the identification of lncRNAs. What’s more, our RF model just needs the training samples of human beings.

### Comparison with other methods

In this paper, we compare the LncRNApred with Coding Potential Calculator (CPC). CPC can distinguish coding from noncoding transcripts with high accuracy by using Support Vector Machine (SVM) based on six biologically meaningful sequence features. The feature set includes three ORF features (LOG-ODDS SCORE, COVERAGE OF THE PREDICTED ORF, INTEGRITY OF THE PREDICTED ORF) and three sequence alignment features (NUMBER OF HITS, HIT SCORE, FRAME SCORE). In order to compare these two methods, we use the same test dataset which includes 28707 lncRNAs and 36198 mRNAs of human, 35373 lncRNAs and 27728 mRNAs of mouse, 2113 lncRNAs of other species. As shown in Table 4, LncRNApred demonstrates the best performance measured by MCC followed by CPC. While LncRNApred and CPC are applied on human dataset, the values of MCC are 0.8569 and 0.7687 respectively. When LncRNApred and CPC are applied on mouse dataset, the values of MCC are 0.8880 and 0.7520 respectively. Additionally, LncRNApred shows the highest specificity compared to CPC. Although the LncRNApred displays a lower sensitivity, CPC shows a higher false positive rate. A lot of lncRNAs are predicted to be the mRNAs by using CPC.

**Table 4:** The performance of CPC.

Species	Positive (lncRNAs)	Negative (mRNAs)	Sn	Sp	ACC	MCC
Human	28707	36198	76.35 (21031/28707)	99.2 (36062/36198)	87.7	0.7687
Mouse	35851	27728	75.27 (26986/35851)	99.8 (27647/27728)	82.5	0.7520
Other species	2113	0	93.3 (1971/2113)	0	93.3	0

### Web implementation

**Fig 10:**
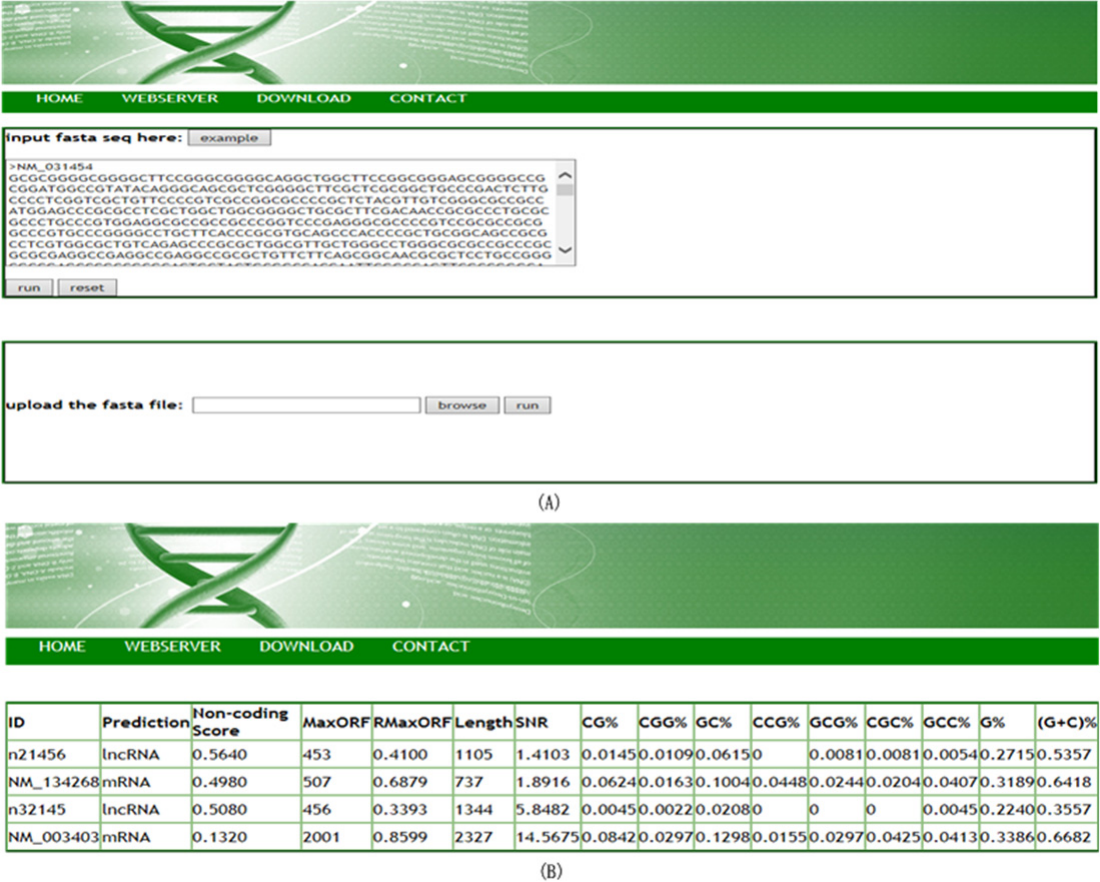
Screenshots of LncRNApred web server. (A) The input page. Single sequence or a fasta file can be as the input of LncRNApred. (B) The output page. LncRNApred reports sequence ID, Non-coding score, predicted class and the information of features.

In this paper, we develop a user-friendly web server named LncRNApred. It is available for free at http://mm20132014.wicp.net:57203/LncRNApred/home.jsp (Fig 10). LncRNApred provides trained RF model based on the training data of human beings. The input of LncRNApred can be a sequence or a fasta file (Fig 10A). The output include sequence ID, Non-coding score, predicted result and the information of features (Fig 10B).

## Conclusion

Identification of lncRNAs is the first step to understand the various of regulatory mechanisms. In this paper, we introduce three new features, including MaxORF, RMaxORF and SNR. A new hybrid feature with 89 dimension can be formed by combining 86 sequence features and the above 3 features together. However, not every feature contribute to the classification accuracy. So we optimize the 89 dimensional features using the feature score criterion (FSC). The first 30 features of FSC are selected as the input vector of the classifier. Besides, an RF classifier model is constructed to discover new lncRNAs. Robustness is an advantage of RF model, since it can be used to build the ensemble of trees by randomly selecting features. The accuracy of a RF classifier is highly depends on the selection of training samples. In order to choose representative samples to construct training dataset, we use Self Organizing Feature Map (SOM) to select the training dataset. Finally, we provide a highly reliable and accurate tool called LncRNApred. It can identify the lncRNAs from thousands of assembled transcripts accurately andquickly. Moreover, using LncRNApred, we can also predict protein-coding potential of transcripts. The results indicate that our LncRNApred outperforms CPC. Therefore, we believe that V-ELMpiRNAPred is a valuable tool for the study of lncRNA and protein-coding transcripts.

## Supporting Information

S1 Table The positive training data of LncRNApred. The 2033 human lncRNAs are selected as the positive training data.
(RAR)

S2 Table The negative training data of LncRNApred. 2031 human mRNAs are selected as the negative training data.
(RAR)

S3 Table The positive test data (human) of LncRNApred. The 28707 mouse lncRNAs are selected as the positive test data.
(RAR)

S4 Table The negative test (human) data of LncRNApred. The 36198 human mRNAs are selected as the negative test data.
(RAR)

S5 Table The positive test data (mouse) of LncRNApred. The 35851 mouse lncRNAs are selected as the positive test data.
(RAR)

S6 Table The negative test data (mouse) of LncRNApred. The 27728 mouse mRNAs are selected as the negative test data.
(RAR)
